# Antimicrobial Photodynamic Therapy Associated With Systemic Treatment in Osteoradionecrosis: An In Vivo Study

**DOI:** 10.1002/lsm.70163

**Published:** 2026-06-16

**Authors:** Larissa Lopes Fonseca, Lorena dos Reis Pereira Queiroz, Andréia Luiza Oliveira Costa, Cristina Paixão Durães, Renata Sousa Leite, Ludmila Martins Delazeri, Sérgio Henrique Sousa Santos, Alfredo Maurício Batista de Paula, Lucyana Conceição Farias, André Luiz Sena Guimarães

**Affiliations:** ^1^ Department of Dentistry Montes Claros State University (Unimontes) Montes Claros Minas Gerais Brazil; ^2^ Institute of Agricultural Sciences Universidade Federal de Minas Gerais (UFMG) Montes Claros Minas Gerais Brazil

**Keywords:** clindamycin, micro‐CT, osteoradionecrosis, pentoxifylline, photodynamic therapy, tocopherol

## Abstract

**Objective:**

To evaluate the effects of antimicrobial photodynamic therapy (aPDT), systemic pharmacological therapy, and their combination on irradiated mandibular post‐extraction bone repair relevant to osteoradionecrosis (ORN) development in a rat model.

**Methods:**

Twenty‐six male Wistar rats were initially randomized into five experimental groups: non‐irradiated extraction control, irradiated extraction plus systemic pharmacological therapy, irradiated extraction plus combined systemic pharmacological therapy and aPDT, irradiated extraction plus aPDT alone, and irradiated extraction without post‐extraction treatment. The model included a single 20 Gy focal irradiation dose to the left mandible, followed by the first mandibular molar extraction 10 days later. During the experiment, some animals were lost due to procedural causes unrelated to treatment, resulting in final group sizes of 5, 5, 3, 4, and 6, respectively. The results were evaluated by micro‐computed tomography (*p* = 0.02), radiographic examinations, histopathological analysis, and assessment of hepatic steatosis.

**Results:**

Irradiated untreated animals showed the most pronounced structural and histopathological alterations. All treatment protocols improved bone repair compared with the irradiated untreated group. However, the combined protocol consistently showed the most favorable structural, radiographic, and histological findings, with better preservation of bone architecture and more organized osteogenesis.

**Conclusions:**

aPDT combined with systemic pharmacological therapy may represent a promising multimodal strategy for irradiated mandibular bone injury relevant to ORN development.

**Abbreviations**
aPDT: antimicrobial photodynamic therapy
ORN: osteoradionecrosis

## 1 Introduction

Osteoradionecrosis (ORN) is a severe late complication of radiotherapy in the maxillofacial region and may be triggered or aggravated by local trauma, dental extractions, and secondary infection in previously irradiated bone [1]. Its development is multifactorial and influenced by both patient‐ and treatment‐related factors, including smoking, male sex, dental extractions performed before or after radiotherapy, and higher radiation doses [2]. In particular, cumulative radiation doses above 60 Gy are consistently associated with an increased risk of ORN, although dose fractionation remains essential to reduce damage to surrounding normal tissues during treatment [3, 4, 5, 6]. Clinically, ORN may range from limited asymptomatic bone exposure to severe presentations involving pain, secondary infection, trismus, halitosis, fistula formation, sequestration, and pathological fracture, reflecting the progressive and heterogeneous nature of the disease [7, 8]. Because its pathophysiology involves hypovascularity, fibrosis, impaired tissue repair, and frequent microbial contamination, treatment remains challenging and is typically guided by lesion severity and extension [1]. Conservative management may include systemic antibiotics, local antimicrobial measures, hyperbaric oxygen therapy, and pharmacological strategies based on antifibrotic and antioxidant agents, whereas advanced cases often require surgical debridement or resection [1, 9, 10].

Among pharmacological approaches, the combination of pentoxifylline and tocopherol, with or without clodronate, has attracted attention for its potential to improve microcirculation, reduce fibrosis, and support tissue repair in irradiated tissues [9, 10, 11]. In parallel, clindamycin may be a relevant adjunct due to its favorable bone penetration and established utility in the prevention and treatment of bone infections, particularly at contaminated post‐extraction sites within irradiated fields [12, 13].

Adjuvant strategies such as antimicrobial photodynamic therapy (aPDT) with methylene blue have also been explored as complementary approaches because they combine local antimicrobial activity with potential modulation of inflammation and tissue repair [7, 8, 14, 15, 16, 17, 18]. In this context, combining aPDT with systemic pharmacological therapy represents a biologically plausible multimodal strategy for irradiated bone exposed to infection, fibrosis, and impaired repair after dental extraction [9, 10, 14]. Given that tooth extraction is a recognized triggering factor for ORN and that delayed socket healing in irradiated bone may represent an early stage or a condition predisposing to ORN, experimental models based on post‐extraction repair are relevant for evaluating interventions aimed at limiting disease progression and improving bone healing in this context [4, 13, 19, 20].

aPDT reduces microbial burden and inflammation and emerges as an adjuvant treatment for ORN, which is frequently associated with persistent microbial colonization in hypoxic tissues. This hinders the effectiveness of conventional therapies, such as systemic antibiotics, because they have limited penetration into these environments. Therefore, the present study aimed to associate aPDT with existing systemic protocols for ORN, considering a biologically plausible strategy to act synergistically on the different pathogenic mechanisms of the disease.

## 2 Materials and Methods

### 2.1 Ethical Approval and Study Design

This in vivo experimental study was conducted at the Animal Facility of Universidade Estadual de Montes Claros (UNIMONTES), Minas Gerais, Brazil, in accordance with institutional standards for animal care, the ARRIVE 2.0 guidelines, and the recommendations of the Brazilian National Council for the Control of Animal Experimentation (CONCEA). The UNIMONTES Animal Experimentation Ethics Committee approved all procedures under protocol no. 015/2022. The experimental unit consisted of a single animal.

### 2.2 Sample Size, Animals, Randomization, and Experimental Groups

Sample size was estimated a priori using G*Power software (version 3.1.9.7), assuming a significance level of 5% (*α* = 0.05), statistical power of 80%, and an effect size of 0.8, resulting in a minimum required sample of 25 animals [21]. To ensure balanced group allocation and account for possible attrition in this irradiated surgical model, 26 male Wistar rats (*Rattus norvegicus* albinus) were initially included and randomly allocated to five experimental groups. Animals were obtained from the Central Animal Facility of the Institute of Biological Sciences, Universidade Federal de Minas Gerais (UFMG), Minas Gerais, Brazil. At the beginning of the experiment, the rats were approximately 60 days old and weighed between 280 and 350 g. Animals were housed in polysulfone mini‐isolators with autoclaved sawdust bedding, under a 12‐h light/dark cycle and controlled temperature (22°C–25°C), with ad libitum access to food and water. Environmental enrichment was provided, and all animals were monitored daily for general health status and signs of discomfort.

Randomization was performed by assigning cages to groups using an online randomization platform (sorteador.com). The experimental groups and treatment allocation are summarized in Supporting Information S1: 1. Group 1 (G1) consisted of non‐irradiated animals subjected only to dental extraction and served as the negative control for physiological bone healing. Group 2 (G2) included irradiated animals treated with systemic pharmacological therapy consisting of clindamycin, pentoxifylline, and tocopherol. Group 3 (G3) consisted of irradiated animals treated with systemic pharmacological therapy combined with aPDT. Group 4 (G4) included irradiated animals treated exclusively with aPDT. Group 5 (G5) included irradiated animals subjected to dental extraction without additional treatment and served as the positive control for irradiated post‐extraction bone injury relevant to ORN development. A total of 26 animals were initially included in the study and allocated into five experimental groups. During the experimental period, three animals were lost due to anesthetic complications unrelated to the experimental treatments or the aPDT protocol, representing risks inherent to experimental animal procedures. Specifically, one animal from group G4 and two animals from group G3 were lost. Consequently, the final sample consisted of five animals in G1, five in G2, three in G3, four in G4, and six in G5. All remaining animals were included in the final analyses.

### 2.3 Irradiation Protocol

Initially, animals assigned to the irradiated groups underwent radiotherapy under general anesthesia induced by intraperitoneal ketamine (100 mg/kg) (Dopalen, Ceva Saúde Animal, Paulínia, SP, Brazil) and xylazine (10 mg/kg) (Rompun, Bayer SA, São Paulo, SP, Brazil). Irradiation was performed using a linear accelerator (ELEKTA PRECISE, Elekta AB, Stockholm, Sweden; serial number 152493), which emits collimated photon and electron beams. A single focal dose of 20 Gy was delivered to the left mandibular region, with a focus‐to‐skin distance of 100 cm, a depth of 3.5 cm, and a collimation field of 20 × 1 cm, allowing six animals to be irradiated simultaneously under standardized geometry. This protocol followed previously described preclinical models of irradiated mandibular bone injury relevant to the development of ORN. It was selected as a practical experimental strategy, since fractionated irradiation in small animals would require repeated anesthesia and repositioning, potentially compromising the reproducibility of focal mandibular targeting [19, 20]. Irradiation was restricted to the head and neck region, targeting the left mandible while minimizing exposure of adjacent structures [19, 20].

### 2.4 Tooth Extraction Procedure

Ten days after irradiation, the left mandibular first molar was extracted under general anesthesia, as dental extraction is a recognized precipitating factor for ORN in irradiated bone [20, 22]. Extractions were performed by three surgeons using small forceps. The procedure consisted of syndesmotomy, followed by luxation and avulsion, with careful avoidance of residual root fragments and trauma to adjacent bone. Postoperative analgesia was provided with ketoprofen (20 mg/mL) at a volume of 40 µL per animal by subcutaneous administration, corresponding to three daily doses according to CONCEA recommendations.

### 2.5 Post‐Extraction Treatment Protocols

Treatment was initiated 21 days after tooth extraction and maintained for 15 days [20]. The systemic pharmacological protocol was designed to correspond to cumulative human daily doses of pentoxifylline (400 mg) (Pentoxifilina, EMS S.A., Hortolândia, SP, Brazil), clindamycin (300 mg) (Cloridrato de Clindamicina, Teuto Brasileiro S.A., Anápolis, GO, Brazil), and tocopherol (total daily equivalent: 1200 IU; equivalent to three 400 IU capsules) (Vitamina E 400 IU, OficialFarma, Brazil), with doses adjusted according to the body weight of each animal. To reduce repeated handling stress, the equivalent of seven consecutive human daily doses was concentrated and administered once weekly by oral gavage. The dose was individualized for each animal based on body weight and diluted to a final volume of 600 µL in water, with tocopherol prepared as a 3% aqueous solution.

aPDT was performed in the extraction area using 0.01% methylene blue gel (Minas Brasil Laboratory, Minas Gerais, Brazil) associated with a diode laser device (MMOptics, MMOptics Ltda, São Carlos, SP, Brazil; RRID: SCR_015955; GaAlAs/InGaAlP), with a wavelength of 660 nm and an energy delivery of 9 J, according to previously described photodynamic therapy parameters [8]. The aPDT protocol was performed four times during the 15‐day treatment period, on experimental days 0, 2, 7, and 9, for a total of 4 sessions. The intervals between sessions ranged from 2 to 5 days. Accordingly, G1 underwent dental extraction without irradiation or post‐extraction treatment; G2 received systemic clindamycin, pentoxifylline, and tocopherol after irradiation and extraction; G3 received the same systemic pharmacological protocol combined with local aPDT; G4 received local aPDT only after irradiation and extraction; and G5 underwent irradiation and extraction without any post‐extraction treatment, serving as the irradiated positive control.

### 2.6 Euthanasia and Tissue Collection

At the end of the treatment period, animals were euthanized according to a protocol adapted to allow adequate assessment of post‐extraction bone repair in irradiated bone [19, 23]. Sedation was induced by intraperitoneal administration of 2% xylazine (10 mg/kg) (Xylazine, Syntec, São Paulo, SP, Brazil) and 10% ketamine (75 mg/kg) (Ketamine, Syntec, São Paulo, SP, Brazil). Subsequently, propofol was administered by intracardiac injection, and death was confirmed by cardiac auscultation according to CONCEA recommendations. The left hemimandible and liver were collected and fixed in 10% neutral buffered paraformaldehyde.

### 2.7 Micro‐CT Analysis

Bone specimens were analyzed by micro‐computed tomography (micro‐CT) according to validated protocols for rodent alveolar bone evaluation [24, 25]. Microarchitectural parameters were selected in accordance with standardized methodological guidelines for rodent bone microstructure analysis [26] (Supporting Information S2: 2), including bone volume fraction (BV/TV), trabecular thickness (Tb.Th), trabecular number (Tb.N), trabecular separation (Tb.Sp), and total porosity [Po(tot)].

### 2.8 Histological Processing and Outcome Assessment

After micro‐CT acquisition, bone samples were decalcified in a standard decalcifying solution and processed histologically according to validated protocols for decalcified bone tissue [27, 28, 29]. The slides were digitized using a Motic Easy Scan Infinity 60 optical scanning microscope (Motic, Xiamen, Fujian, China). Liver sections were quantified using ImageJ software, and bone sections were analyzed using CaseViewer software. Outcome assessment was performed under blinded conditions, as the examiner responsible for the measurements was unaware of group allocation.

### 2.9 Statistical Analysis

Statistical analyses were performed using PASW Statistics 26 (IBM, Armonk, NY, USA). Data are presented as mean ± standard error (SE). Normality was assessed using the Shapiro–Wilk test. Group differences were analyzed using one‐way analysis of variance (ANOVA), followed by Tukey's post hoc test. A significance level of *p* < 0.05 was adopted. Graphs were generated using GraphPad Prism 10 (GraphPad Software, San Diego, CA, USA).

## 3 Results

**Figure 1:**
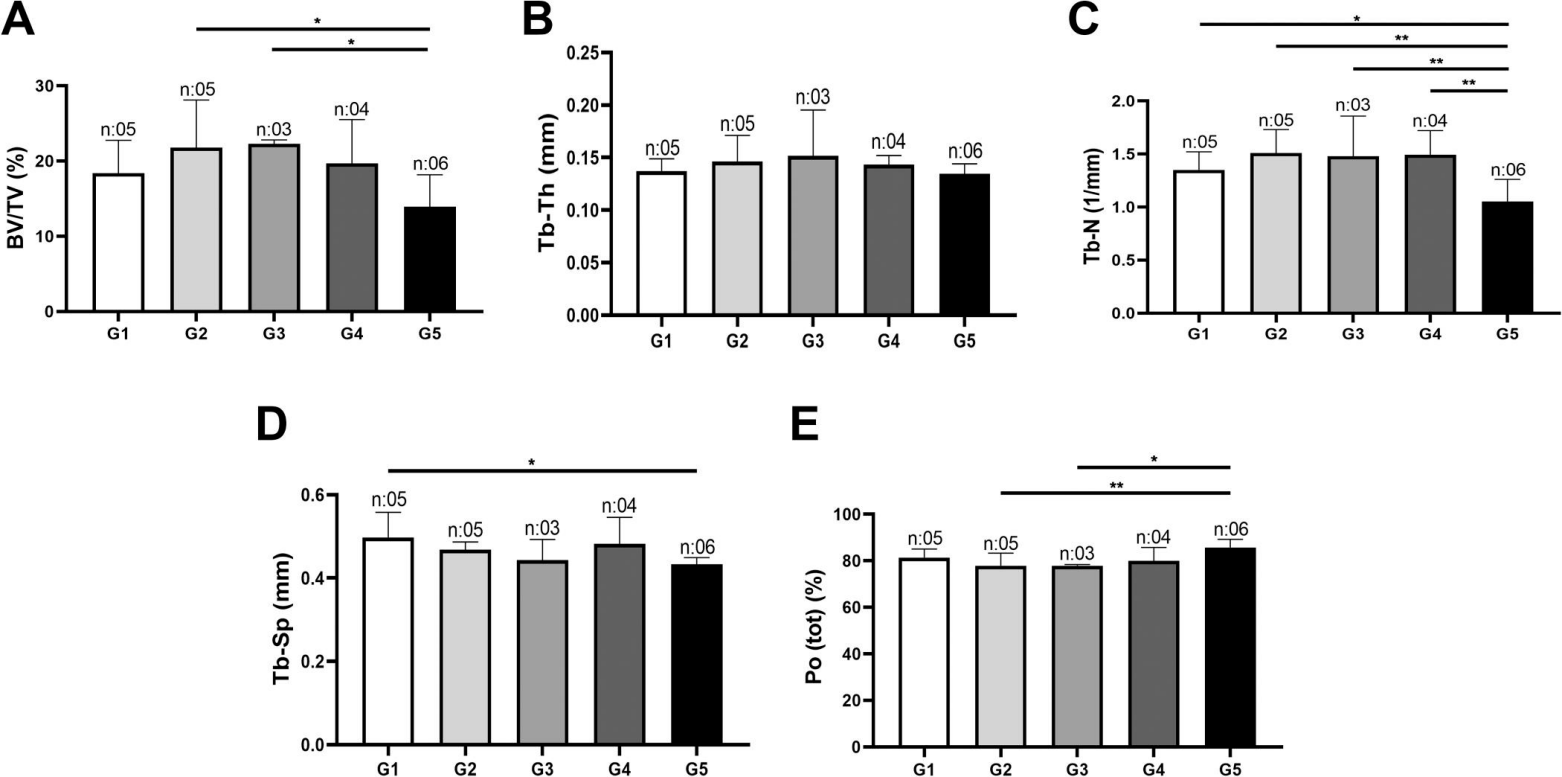
Quantitative micro‐CT analysis of alveolar bone microarchitecture. Quantitative micro‐computed tomography (micro‐CT) analysis of alveolar bone microarchitecture across the experimental groups. (A) Bone volume fraction (BV/TV, %); (B) trabecular thickness (Tb.Th, mm); (C) trabecular number (Tb.N, mm); (D) trabecular separation (Tb.Sp, mm); and (E) total porosity [Po(tot), %]. G1: non‐irradiated extraction control; G2: irradiated + extraction + systemic pharmacological treatment; G3: irradiated + extraction + systemic pharmacological treatment combined with antimicrobial photodynamic therapy (aPDT); G4: irradiated + extraction + aPDT alone; and G5: irradiated + extraction without post‐extraction treatment. Data are expressed as mean ± standard error of the mean (SEM). The number of animals analyzed in each group is indicated above each bar. Horizontal bars indicate statistically significant intergroup comparisons. Asterisks denote statistically significant differences (*p < 0.05; **p < 0.005).

Micro‐computed tomography (micro‐CT) quantitative analysis demonstrated significant differences in alveolar bone microarchitecture among the experimental groups (Figure 1). The inclusion of the non‐irradiated extraction control group (G1) allowed a clearer comparison with the irradiated untreated control group (G5). G5 showed a significant reduction in trabecular number (Tb.N) compared with G1 and with all treated irradiated groups (G2–G4), indicating impaired trabecular organization (Figure 1C). G5 also showed significantly lower trabecular separation (Tb.Sp) than G1 (Figure 1D). Regarding bone volume fraction (BV/TV), G2 and G3 showed significantly higher values than G5, indicating better preservation of alveolar bone volume (Figure 1A). Total porosity [Po(tot)] was significantly higher in G5 than in G2 and G3, further supporting the protective effect of systemic therapy, especially when associated with aPDT (Figure 1E). Trabecular thickness (Tb.Th) did not differ significantly among groups (Figure 1B). Overall, these quantitative findings indicate that irradiation followed by tooth extraction impaired alveolar bone microarchitecture, while the therapeutic interventions attenuated radiation‐induced bone deterioration. The evaluated micro‐CT parameters are detailed in Supporting Information S2: 2.

**Figure 2:**
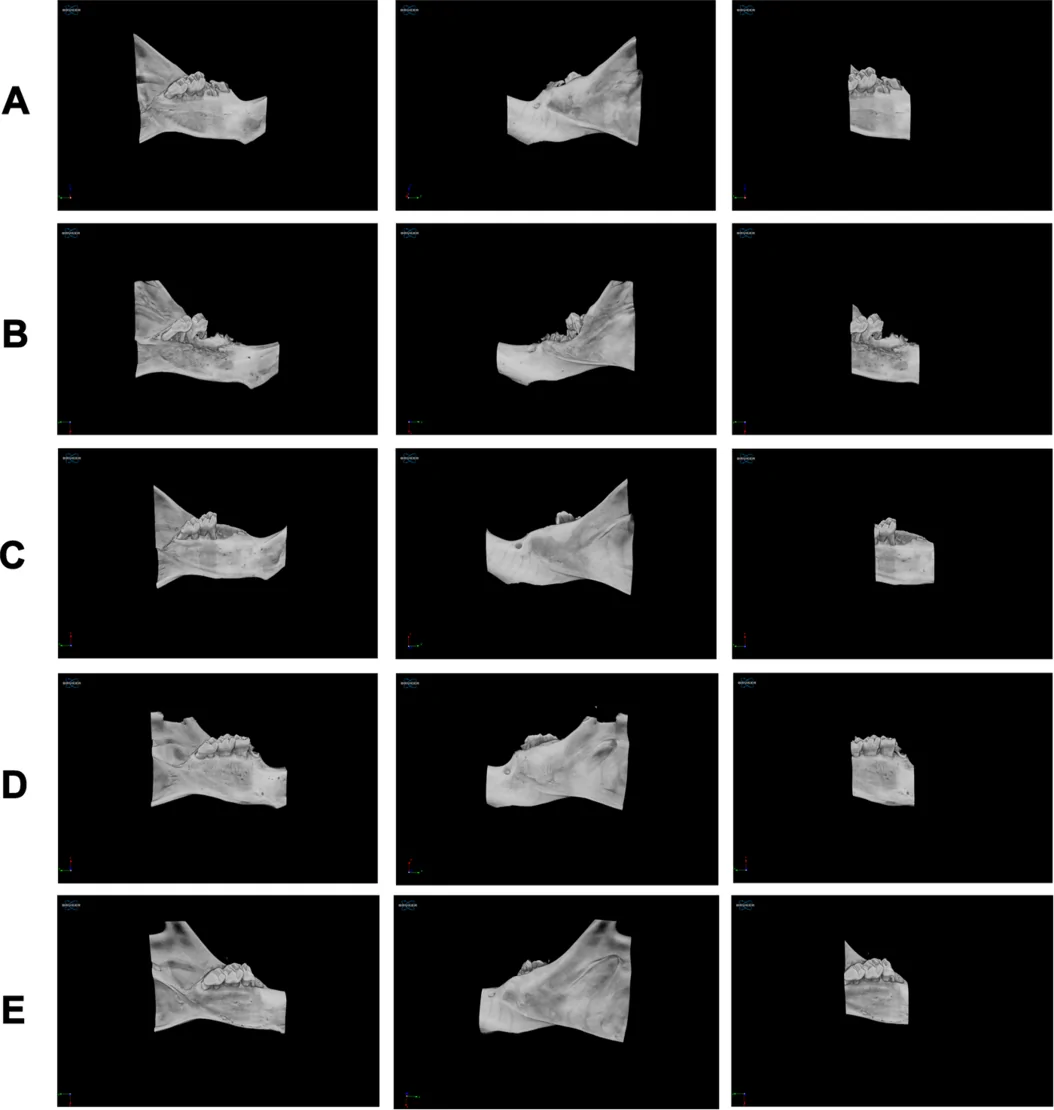
Representative three‐dimensional micro‐CT reconstructions of mandibular bone healing across the experimental groups. Representative three‐dimensional micro‐computed tomography (micro‐CT) reconstructions of rat mandibles obtained after tooth extraction in the experimental groups. Images were acquired using a SkyScan 1174 microtomography system (Bruker, Kontich, Belgium). (A) Group 1 (G1): non‐irradiated extraction control; (B) Group 5 (G5): irradiated positive control (single 20 Gy dose + extraction, no treatment); (C) Group 2 (G2): irradiated + extraction + systemic treatment with clindamycin (300 mg), pentoxifylline (400 mg), and tocopherol (1200 IU); (D) Group 3 (G3): irradiated + extraction + antimicrobial photodynamic therapy (aPDT) using 0.01% methylene blue gel combined with systemic treatment with clindamycin (300 mg), pentoxifylline (400 mg), and tocopherol (1200 IU); and (E) Group 4 (G4): irradiated + extraction + aPDT using 0.01% methylene blue gel. G5 exhibited the most severe bone deterioration, characterized by reduced radiodensity, structural disorganization, and increased porosity, supporting the validity of the experimental model relevant to osteoradionecrosis development. In contrast, G1 showed preserved and homogeneous bone architecture, consistent with physiological post‐extraction healing.

Qualitative analysis of the three‐dimensional micro‐CT reconstructions corroborated the quantitative findings. The irradiated untreated control group (G5) exhibited reduced segmented bone volume, lower radiodensity, trabecular disorganization, and increased porosity, consistent with impaired post‐extraction bone healing in irradiated bone, a condition relevant to ORN development (Figure 2B). In contrast, all treated irradiated groups (G2–G4) showed attenuation of these necrotic changes to varying degrees (Figure 2C–E). G2, which received systemic treatment with clindamycin, pentoxifylline, and tocopherol, showed partial preservation of bone integrity compared with G5 (Figure 2C). The most favorable qualitative outcome was observed in G3, which received combined systemic treatment and aPDT, and demonstrated the best‐preserved bone architecture, highest radiodensity, and greatest structural organization among all irradiated groups (Figure 2D). G4, treated with aPDT alone, also showed improved bone quality relative to G5, although to a lesser extent than G3 (Figure 2E). These findings suggest a synergistic effect of aPDT when associated with systemic pharmacological therapy.

**Figure 3:**
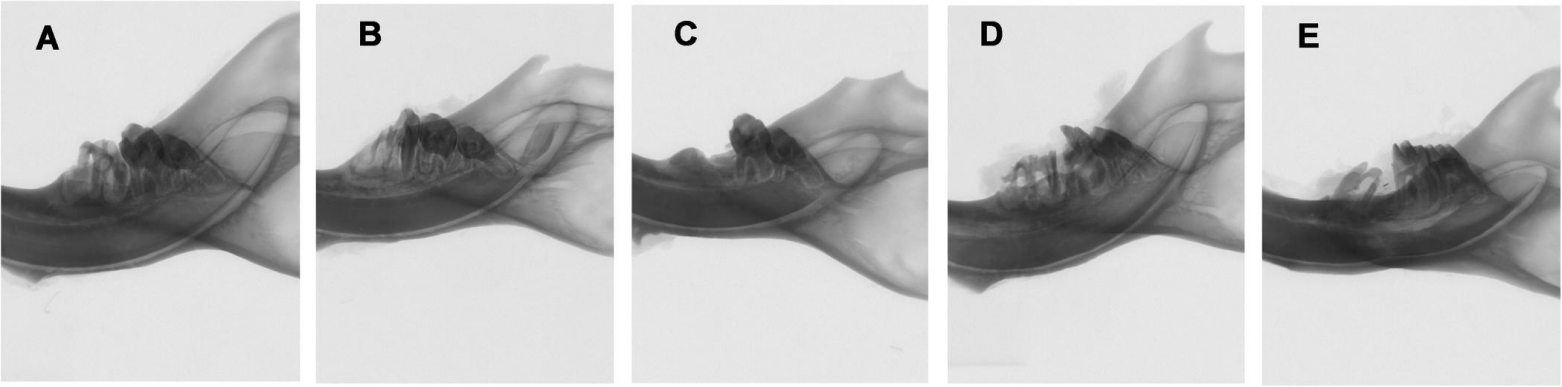
Representative radiographic evaluation of mandibular bone repair in the experimental groups. Representative lateral radiographic images of rat mandibles showing post‐extraction bone repair in the experimental groups. (A) Group 1 (G1): non‐irradiated extraction control; (B) Group 5 (G5): irradiated positive control (single 20 Gy dose + extraction, no treatment); (C) Group 2 (G2): irradiated + extraction + systemic treatment with clindamycin (300 mg), pentoxifylline (400 mg), and tocopherol (1200 IU); (D) Group 3 (G3): irradiated + extraction + aPDT using 0.01% methylene blue gel combined with systemic treatment with clindamycin (300 mg), pentoxifylline (400 mg), and tocopherol (1200 IU); and (E) Group 4 (G4): irradiated + extraction + aPDT using 0.01% methylene blue gel.

Radiographic evaluation further supported the micro‐CT findings. The irradiated untreated control group (G5) showed marked impairment of post‐extraction bone consolidation, with radiographic features compatible with ORN (Figure 3B). In contrast, G2 exhibited improved bone repair compared with G5, with greater radiographic evidence of bone formation and structural continuity (Figure 3C). The most pronounced reversal of radiation‐induced damage was observed in G3, which showed denser and more organized bone repair, consistent with superior preservation of mandibular architecture (Figure 3D). G4 also demonstrated improved bone consolidation relative to the irradiated untreated group, reinforcing the beneficial effect of local aPDT, although the combined protocol remained the most favorable therapeutic strategy (Figure 3E). Collectively, the imaging findings indicate that all treatment protocols improved bone repair compared with the irradiated untreated control, with the combination of systemic therapy and aPDT providing the best overall response.

**Figure 4:**
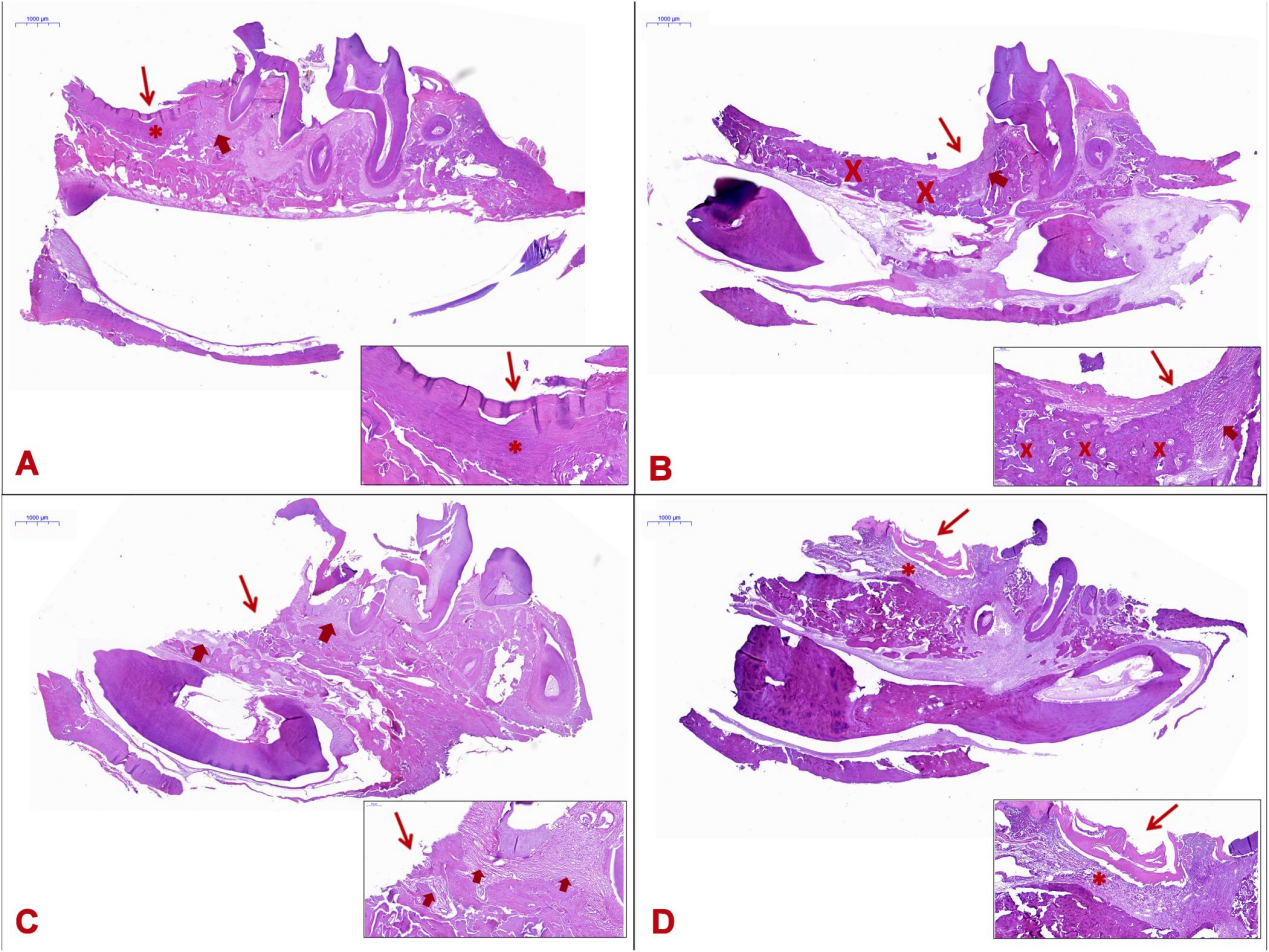
Histological evaluation of post‐extraction alveolar socket repair in the experimental groups. Representative histological sections of post‐extraction alveolar sockets from the experimental groups were stained with hematoxylin and eosin (H&E) and examined under light microscopy. Scale bars: main images = 1000 µm; insets = 100 µm. Thin arrows indicate the extraction socket; thick arrows indicate the region of interest for repair analysis; arrowheads indicate newly formed bone; asterisks (*) indicate inflammatory infiltrate; and X indicates necrotic areas. (A) Group 1 (G1): non‐irradiated control, showing physiological bone healing with organized newly formed bone filling the socket; (B) Group 2 (G2): irradiated + systemic treatment, showing partial bone repair with fibrous connective tissue and residual necrotic areas; (C) Group 3 (G3): irradiated + systemic treatment + adjunctive aPDT, showing the most organized and dense bone formation among irradiated groups; and (D) Group 4 (G4): irradiated + local aPDT, showing new bone formation associated with a more evident inflammatory infiltrate.

Histological analysis of the post‐extraction alveolar sockets confirmed the imaging findings. In the non‐irradiated control group (G1), physiological repair was observed, characterized by organized granulation tissue, deposition of newly formed bone trabeculae, and preservation of the surrounding soft tissue architecture, without excessive fibrosis, necrosis, or hypovascularization (Figure 4A). In G2, the repair process was partially restored but remained heterogeneous, with irregular new bone formation, residual fibrous connective tissue, and incomplete structural organization compared with G1 (Figure 4B). Among the irradiated groups, G3 showed the most favorable histological repair pattern, with more advanced and better‐organized osteogenesis, reduced fibrous connective tissue deposition, and less chronic inflammatory infiltrate, resulting in a healing pattern closer to that observed in the physiological control group (Figure 4C). G4 also demonstrated new bone formation, but with a more intermediate pattern characterized by intercalated fibrous connective tissue and less organization than G3 (Figure 4D). Histologically, these results reinforce that aPDT alone exerted a beneficial local effect. Still, the combination of aPDT with systemic pharmacological treatment yielded the most consistent improvement in post‐radiation alveolar repair.

**Figure 5:**
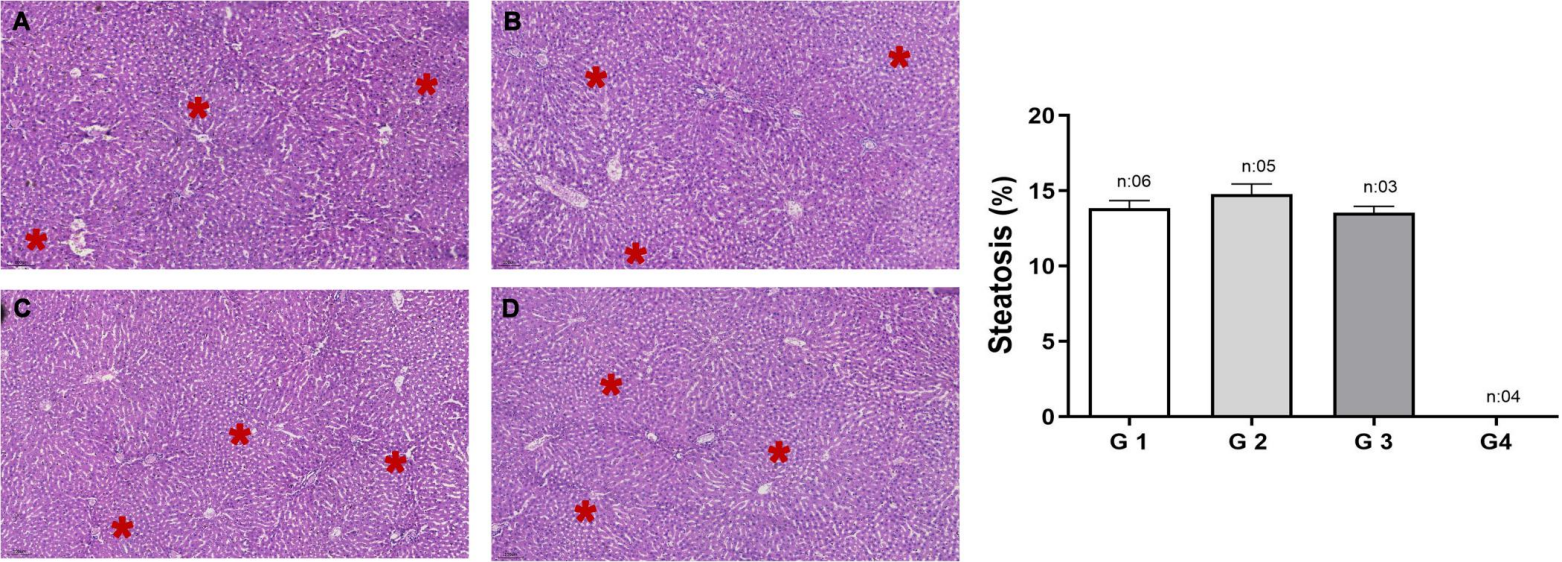
Histological assessment of liver tissue and quantitative analysis of hepatic steatosis. Representative histological images of liver tissue and quantitative analysis of hepatic steatosis in the experimental groups. Liver sections were stained with hematoxylin and eosin (H&E) and examined under light microscopy (original magnification ×100). Scale bar = 100 µm. Asterisks (*) indicate representative areas of hepatic steatosis. (A) Group 1 (G1): non‐irradiated control; (B) Group 2 (G2): irradiated + systemic treatment with clindamycin, pentoxifylline, and tocopherol; (C) Group 3 (G3): irradiated + aPDT combined with systemic treatment; and (D) Group 4 (G4): irradiated + aPDT using 0.01% methylene blue gel. The bar graph shows the percentage of hepatic steatosis quantified according to the scoring system described by Kleiner et al. [30]. Data are presented as mean ± standard error of the mean (SEM), and the number of animals analyzed (*n*) is indicated above each bar. Although G4 showed lower steatosis values, no statistically significant differences were observed among the experimental groups (*p* > 0.05).

To assess potential systemic toxicity, hepatic steatosis was evaluated histologically using the previously described scoring system [30]. Groups G1, G2, and G3 showed comparable levels of steatosis, ranging from approximately 13% to 15%, with overlapping error bars and no statistically significant differences among them (Figure 5A–C). G1, which was not irradiated or treated, presented a baseline steatosis value of approximately 14%. At the same time, G2 and G3 showed similar values, suggesting that systemic treatment alone or in combination with aPDT did not increase hepatic lipid accumulation relative to baseline. Interestingly, G4 exhibited the lowest steatosis level among all groups, with residual values close to zero (approximately 0.01%) (Figure 5D). However, despite this apparent reduction, no statistically significant differences were observed among the experimental groups. Overall, the liver findings suggest that the tested therapeutic protocols were not associated with relevant hepatic toxicity under the conditions of this study.

Taken together, these findings indicate that all active interventions improved bone repair compared with the irradiated untreated group. At the same time, the combination of aPDT with systemic pharmacological therapy produced the most consistent improvements across micro‐CT, radiographic, and histopathological analyses, representing the most promising strategy for improving irradiated mandibular post‐extraction bone repair in this experimental model relevant to ORN development.

## 4 Discussion

The combined protocol produced the most favorable outcomes across micro‐CT, radiographic, and histological analyses, suggesting that adjunctive aPDT may enhance the effects of systemic therapy in irradiated bone [19, 20]. Experimental evidence indicates that ORN induction depends on radiation dose, extraction timing, and overall protocol aggressiveness [4, 19, 22, 31]. A single dose of 50 Gy combined with molar extraction has been shown to reliably induce ORN, whereas 35 Gy appears less effective [31]. Conversely, 30 Gy brachytherapy followed by extraction on the next day caused substantial animal morbidity, highlighting the limitations of overly aggressive protocols [22]. Additional in vivo data showed that single doses of 14–20 Gy are sufficient to induce progressive bone alterations, with fibrosis becoming evident within 2–3 weeks after irradiation and more severe changes occurring at higher doses [19]. Delayed extraction also appears critical, as a 10‐day interval after irradiation has been proposed as a suitable balance between recovery and procedural safety [20]. Within this context, the 20 Gy model used in the present study appears both reproducible and biologically relevant for evaluating therapeutic response [19, 20].

Micro‐computed tomography (micro‐CT) was central to the structural evaluation, allowing objective assessment of alveolar bone microarchitecture [26]. The selected parameters, based on established rodent bone micro‐CT standards, showed better preservation of bone structure in the treated groups than in the irradiated, untreated control, with the most favorable pattern observed in the combined therapy group [24, 25, 26]. These findings are consistent with prior studies supporting micro‐CT as a reliable tool for assessing radiation‐induced changes in jawbone structure [23, 26]. The three‐dimensional reconstructions further corroborated the quantitative results by demonstrating marked trabecular disorganization and porosity in the irradiated untreated group, with visible attenuation of these alterations after treatment [19, 23]. The systemic regimen also has a plausible biological basis [9, 10, 11, 23]. Pentoxifylline and tocopherol have been proposed as antifibrotic and microcirculatory agents in irradiated tissues, and previous clinical studies have reported favorable outcomes in ORN management using these agents, including the PENTOCLO concept in more advanced cases [9, 10]. Experimental studies in irradiated jaw models likewise suggest that pentoxifylline and tocopherol may improve tissue repair and reduce radiation‐related damage [19, 23].

In the present study, clindamycin may also have contributed by reducing microbial burden in the post‐extraction environment. However, the individual contribution of each drug cannot be isolated within the current design [12, 19]. The contribution of aPDT deserves particular emphasis [8, 14, 17]. Although aPDT alone improved bone repair compared with the irradiated untreated control, the best outcomes were consistently observed when it was combined with systemic therapy [20, 32]. This pattern suggests a synergistic interaction between local and systemic mechanisms [32, 33, 34]. Such an interpretation is biologically plausible, as aPDT has been associated with antimicrobial activity, modulation of inflammation, and improved local healing in oral tissues [8, 14]. Clinical reports have also suggested that laser‐based adjuvant approaches [35], including aPDT, may be useful in ORN management when incorporated into multimodal protocols [15]. Ferigatto et al. demonstrated that aPDT, when used as an adjunct to surgical debridement and local antiseptics in the management of ORN, promoted mucosal healing and controlled disease progression in 80% of cases [36]. This interpretation is consistent with the pathophysiology of ORN, which involves hypovascularity, hypocellularity, fibrosis, chronic inflammation, and frequent secondary microbial colonization [2]. Therefore, combining a local therapy aimed at microbial and inflammatory control with a systemic regimen targeting fibrosis and vascular impairment may provide a broader therapeutic effect than isolated interventions [11, 14]. This view is aligned with current perspectives on individualized and multimodal ORN management, particularly in more advanced or persistent cases [13]. No significant differences in hepatic steatosis were observed among groups, as assessed by histological scoring according to established criteria [30]. Although exploratory, these findings suggest that the tested regimens were not associated with evident short‐term hepatic alterations under the present experimental conditions [30]. This study is limited by the small number of animals, the lack of molecular analyses, and the inherent constraints of extrapolating experimental findings to clinical ORN.

Nonetheless, the consistency of the imaging and histological findings supports the robustness of the model and justifies further investigation of the combined protocol in future preclinical and translational studies [19, 23].

## 5 Conclusions

The combination of aPDT with systemic pharmacological therapy may represent a promising strategy for the management of ORN, as it consistently improved structural, radiographic, and histopathological parameters in this experimental model.

## Ethics Statement

All applicable international, national, and/or institutional guidelines for the care and use of animals were followed. The experimental protocol was approved by the Ethics Committee on Animal Use (CEUA) of Universidade Estadual de Montes Claros (protocol no. 015/2022).

## Conflicts of Interest

A.L.S.G., L.C.F., and A.M.B.P. may have potential intellectual or proprietary interests related to the technique evaluated. To minimize potential bias, these authors were not involved in key methodological procedures, data analysis, or interpretation of the results related to the potentially affected components. Independent oversight was implemented to ensure scientific rigor and objectivity. The authors declare no conflicts of interest.

## Supporting information

Supporting File 1

Supporting File 2

## Data Availability

The data that support the findings of this study are available from the corresponding author upon reasonable request.
