# Supplementary material for: Antimicrobial Photodynamic Therapy Associated With Systemic Treatment in Osteoradionecrosis: An In Vivo Study
Source: Lasers Surg Med. 2026 Jun 16;58(8):683–92. doi: 10.1002/lsm.70163 (PMC13580414; doi:10.1002/lsm.70163)
Supplement: Supplementary file 1 [file LSM-58-683-s001.docx]

**Supplementary Material 1.** Experimental Group Allocation and Treatment Protocol.

| **Code** | **Irradiation Status** | **Post-Extraction Treatment Protocol** | **n** | **Primary Objective** |
| --- | --- | --- | --- | --- |
| **Group 1 (G1)** | Non-Irradiated | Dental Extraction Control | 5 | Serve as the negative Control for normal bone healing without Osteoradionecrosis (ORN) compromise. |
| **Group 5 (G5)** | Irradiation (Single 20Gy Dose) | Dental Extraction,  No Treatment | 6 | Positive Control for the induction and development of ORN. |
| **Group 2 (G2)** | Irradiation (Single 20Gy Dose) | Extraction + Systemic** Clindamycin (300 mg) + Pentoxifylline (400 mg) +  Tocopherol (1200 IU) | 5 | Evaluate the efficacy of systemic PENTO therapy in the prevention/management of ORN. |
| **Group 3 (G3)** | Irradiation (Single 20Gy Dose) | Extraction + aPDT #(0.01% Methylene Blue Gel) + Systemic** Clindamycin (300 mg) + Pentoxifylline (400 mg) +  Tocopherol (1200 IU) | 5 | Assess the synergistic effect of systemic PENTO therapy combined with local Antimicrobial Photodynamic Therapy (aPDT). |
| **Group 4 (G4)** | Irradiation (Single 20Gy Dose) | Extraction + aPDT  #(0.01% Methylene Blue Gel) | 5 | Evaluate the efficacy of local aPDT as a standalone treatment for ORN. |
| **Group 5 (G5)** | Irradiation (single 20 Gy dose) | Dental extraction without post-extraction treatment (positive Control) | 6 | G5 served as the irradiated positive Control for post-extraction bone injury relevant to osteoradionecrosis development. |

**Supplementary Material 2**. Micro-CT parameters used for quantitative evaluation of bone microarchitecture.

| **Variable** | **Abbreviation** | **Description** |
| --- | --- | --- |
| **Bone volume fraction** | BV/TV (%) | Ratio of the segmented bone volume to the total volume of the region of interest. |
| **Trabecular thickness** | Tb-Th (mm) | Mean thickness of trabeculae, assessed using direct 3D methods. |
| **Trabecular number** | Tb-N (1/mm) | Measure of the average number of trabeculae per unit length. |
| **Trabecular separation** | Tb-Sp (mm) | Mean distance between trabeculae, assessed using direct 3D methods. |
| **Total porosity** | Po(tot) (%) | The total porosity (expressed as a percentage) is defined as the ratio between the pore volume, representing the void space within the material, and the total volume of the specimen, which comprises the combined volume of the solid phase and the pore spaces. The resulting ratio is then multiplied by 100 to obtain the porosity in percentage terms. |
